# Mitophagy in neurological disorders

**DOI:** 10.1186/s12974-021-02334-5

**Published:** 2021-12-22

**Authors:** Lijun Zhang, Lei Dai, Deyuan Li

**Affiliations:** 1 Department of Pediatrics, West China Second University Hospital, Sichuan University, Chengdu, 610041 China; 2 Key Laboratory of Birth Defects and Related Disease of Women and Children (Sichuan University), Ministry of Education, Chengdu, 610041 Sichuan China; 3 State Key Laboratory of Biotherapy and Cancer Center, West China Hospital, Sichuan University and Collaborative Innovation Center for Biotherapy, Chengdu, 610041 Sichuan China

**Keywords:** Mitophagy, Autophagy, Neurological diseases, Alzheimer's disease, Huntington's disease, Stroke

## Abstract

Selective autophagy is an evolutionarily conserved mechanism that removes excess protein aggregates and damaged intracellular components. Most eukaryotic cells, including neurons, rely on proficient mitophagy responses to fine-tune the mitochondrial number and preserve energy metabolism. In some circumstances (such as the presence of pathogenic protein oligopolymers and protein mutations), dysfunctional mitophagy leads to nerve degeneration, with age-dependent intracellular accumulation of protein aggregates and dysfunctional organelles, leading to neurodegenerative disease. However, when pathogenic protein oligopolymers, protein mutations, stress, or injury are present, mitophagy prevents the accumulation of damaged mitochondria. Accordingly, mitophagy mediates neuroprotective effects in some forms of neurodegenerative disease (e.g., Alzheimer's disease, Parkinson’s disease, Huntington's disease, and Amyotrophic lateral sclerosis) and acute brain damage (e.g., stroke, hypoxic–ischemic brain injury, epilepsy, and traumatic brain injury). The complex interplay between mitophagy and neurological disorders suggests that targeting mitophagy might be applicable for the treatment of neurodegenerative diseases and acute brain injury. However, due to the complexity of the mitophagy mechanism, mitophagy can be both harmful and beneficial, and future efforts should focus on maximizing its benefits. Here, we discuss the impact of mitophagy on neurological disorders, emphasizing the contrast between the positive and negative effects of mitophagy.

## Background

**Fig. 1.**
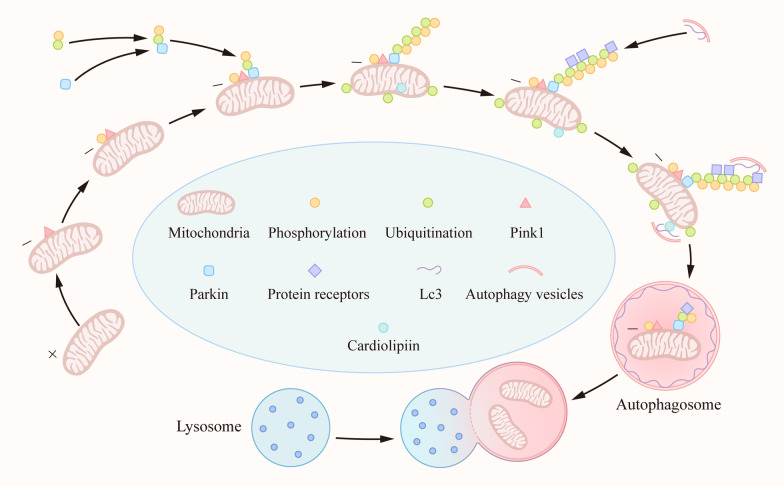
Mechanism of mitophagy. When mitochondria depolarize or accumulate misfolded membrane proteins, PINK1 is located on the outer mitochondrial membrane and phosphorylates itself. At the same time, phosphorylated Ub can directly bind to Parkin and is recruited to the surface of mitochondria and phosphorylated by PINK1. PINK1 can phosphorylate Ub in the whole cell and form polyubiquitin chains. While recruiting Parkin, the mitochondrial matrix is also ubiquitinated. Mitochondria labeled with ubiquitin recruit mitophagy receptors, such as p62, NDP52, NBR1, and OPTN. These receptors interact with the autophagy protein LC3 on the outer membrane through their LIR motifs, bind to the polyubiquitin chain through the ubiquitin binding domain, and finally initiate mitophagy. In addition, cardiolipids in the brain are present in the mitochondrial intimum, requiring three translocases to be exposed to the OMM, and can directly bind to LC3 to mediate mitophagy.

The process of autophagy was discovered using transmission electron microscopy more than 50 years ago [1], and its mechanisms were identified using fluorescent microscopy in 2004 [2]. The following year, Lemasters proposed the term "mitophagy" [3]. Three main types of autophagy have been described: macroautophagy, microautophagy, and chaperone-mediated autophagy (CMA) [4, 5]. Specifically, macroautophagy starts with the formation of a phagophore, generated *de novo* from pre-existing intracellular precursor molecules or multiple sources, which mature into double-membraned vesicles known as autophagosomes [5] (Fig. 1). Several form of macroautophagy have been identified to participate in turnover of damaged organelles, such as mitochondria (mitophagy), ribosomes (ribophagy), and endoplasmic reticulum (ER)-phagy. In contrast, microautophagy begins with the isolation of the cargo, engulfed by the direct invagination of the lysosomal membrane [4]. Finally, CMA depends on the recognition of autophagic substrates bearing a KFERQ motif by cytosolic chaperones of the heat–shock protein family, followed by lysosome-associated membrane protein2 (LAMP2)-dependent translocation of these substrates across the lysosomal membrane [5]. A pentapeptide motif biochemically related to KFERQ is identified as a binding site for a cytosolic chaperone [6]. This motif consist of an invariant amino acid, a glutamine (Gln), at the beginning or end of the sequence, one of the two positively charged amino acids lysine (Lys) or arginine (Arg), one of the four hydrophobic amino acids phenylalanine (Phe), valine (Val), leucine (Leu) or isoleucine (Ile) and one of the two negatively charged amino acids glutamicacid (Glu) or aspartic acid (Asp) [5, 7]. Chaperone-mediated autophagy selectively degrades single proteins, and microautophagy can take up small aggregates and oligomers; however, irreversible aggregates or aberrant oligomeric complexes can only be eliminated by macroautophagy [3]. Mitophagy is the only known pathway through which whole mitochondria can be selectively eliminated.

Next, we will discuss the mechanism of macroautophagy in the nervous system. Autophagy is important for the quality control of cells in the nervous system, as many of these cells are postmitotic cells that cannot dilute protein toxins through mitosis and need exquisite quality control systems in place to eliminate altered proteins and organelles [8]. Although the adult brain only accounts for 2% of the human body weight, it is responsible for 20% of the resting metabolism of the body, and the demand for metabolic energy is strong [9, 10]. These high energy requirements also make the brain susceptible to damage during periods of anoxia or ischema, whether on an acute or chronic basis [11]. Bioenergetic failure has been suggested to cause neuronal death in a range of neurodegenerative diseases [12–15]. Neuronal cells, differ from non-neuronal cells, and rely mainly on the oxidative phosphorylation of mitochondria to produce ATP. Therefore, mitophagy is important in the central nervous system. Numerous studies have confirmed that mitophagy may serve as a targeted treatment for certain diseases. Importantly, the mechanisms of mitophagy are involved in chronic diseases of the nervous system, such as neurodegenerative diseases. However, carfilzomib (also known as PR-171) is a drug for multiple myeloma therapy that irreversibly and covalently binds to two intracellular receptors which are identified as the 20S proteasome catalytic subunits LMP2 and LMP7, respectively [16]. The proteasome can lead to mitophagy defects by degrading BCL/adenovirus E1B interacting protein 3-like (Bnip3L) [17]. Carfilzomib can restore the mitophagy of neurons and prevent acute or chronic ischemic brain injury by reversing Bnip3L degradation, suggesting that mitophagy may not only play a role in chronic diseases of the nervous system [17].

## Main text

### Signaling pathways in mitophagy

Multiple receptor signaling pathways have been confirmed to be involved in mitophagy, and over the past 5 years alone, over 1,800 related articles have been published. Controversy surrounds many of the pathways that have been studied (Table 1).

**Table 1:** Pharmacological regulation of autophagy/mitophagy in neurological diseases

Disease	Drug	Target	Model	Function
AD	UA/AC	AMPK	iPSC neurons	Inhibit mitochondrial fission, increase ATP production ↑
AD	rapamycin	mTOR	PDAPP mice	Inhibits mTOR activity ↑
PD	TFEB	LAMP1	nigra neurons	Induces autophagosome and lysosome biogenesis
HD	PINK1	Parkin	Drosophila	Inhibits accumulation of mitochondria ↑
ALS	Atg1	mTOR	Drosophila	Positively regulates the initiation of autophagy
Stroke	Melatonin	LC3, PINK1	SAH rat	Inhibits dysfunctional mitochondria ↑
Stroke	BNIP3 Silencing	LC3	I/H mice	Inhibits excessive mitophagy
HIE	3-MA	LC3II, p62	HIBI rat	Inhibits autophagy flux
HIE	Glycine	p-AMPK	HI rat	Downregulates AMPK to alleviate autophagy flux ↓
HIE	IPC	p-AMPK	pMCAO rat	Activates AMPK to induce autophagy
HIE	AG	miR-30d-5p	HI rat	Inhibits autophagy^a^
HIE	AT	miR-30d-5p	HI rat	Enhance autophagy
Epilepsy	rapamycin	mTORC	TSC mouse	Inhibits S6 phosphorylation ↑
TBI	Morin	LC3II/I, Beclin-1	TBI rat	Augments autophagy
TBI	NIX	LC3II/I, p62	TBI rat	Increases autophagy
TBI	17-AAG	LC3II, Beclin-1	TBI rat	Induces autophagy
TBI	Wnt3α	β-proteasomal	TBI mouse	Activates Wnt/β-catenin pathway ↑

AD, Alzheimer's disease; UA/AC, urolithinA/actinonin; AMPK, AMP-activated protein kinase; iPSC, induced pluripotent stem cell; ATP, Adenosine Triphosphate; mTOR, mechanistic target of rapamycin complex; PD, Parkinson’s disease; TFEB, transcription factor EB; LAMP1, lysosome-associated membrane protein1; HD, Huntington's disease; PINK1, PTEN-induced putative kinase 1; LC3, light chain 3; SAH, subarachnoid hemorrhage; BNIP3, B-cell lymphoma 2 kilodalton interacting protein 3; I/H, ischemia/hypoxia; HIE, hypoxic–ischemic encephalopathy; 3-MA, 3-methyladenine; HIBI, hypoxic–ischemic brain injury; p-AMPK, phosphorylation of AMPK; HI, hypoxia–ischemia; IPC, ischemic preconditioning; pMCAO, permanent middle cerebral artery osslusion; AG, Agomir; AT, Antagomir; TSC, tuberous sclerosis complex; TBI, Traumatic brain injury

^a^Harmful to disease; ↑ Up-regulate autophagy/mitophagy; ↓ Down-regulate autophagy/mitophagy.

### Protein pathways

#### PINK1/Parkin

Phosphatase and tensin (PTEN)-induced putative kinase (PINK1) belongs to the serine/threonine protein kinase family, and serves as a sensor of mitochondrial quality that can trigger subsequent Parkin-dependent recruitment of light chain 3 (LC-3) [18]. Under normal conditions, protein levels of PINK1 are low as a result of its cleavage by mitochondrial proteases and subsequent degradation via the N-end rule pathway. These low basal levels of PINK1 prevent aberrant mitophagy of healthy mitochondria [19]. In functional mitochondria, PINK1 is transported into the inner mitochondrial membrane (IMM), where it is processed and cleaved by several proteases. Loss of mitochondrion membrane potential, encountering misfolded membrane proteins, or knockout of specific mitochondrial proteins that cleaves PINK1 will cause PINK1 to be stabilized on the outer surface of the mitochondrion, triggering mitophagy. The events downstream of PINK1 stabilization include phosphorylation of Miro and Mitofusin, phosphorylation (and activation) of Parkin (an E3 ubiquitin ligase), and phosphorylation of ubiquitin (p-Ub). Parkin binds to both phospho-Miro and phospho-ubiquitin and is recruited to the mitochondrial outer membrane surface. Phosphorylated Ub (p-Ub) binds directly to a pocket around the H302 residue within Parkin. Through direct binding with p-Ub, the Ubl domain of Parkin becomes accessible, allowing PINK1-mediated phosphorylation. At the same time, PINK1 is free to phosphorylate the free Ub spread throughout the cytoplasm. Parkin mediates a feed-forward mechanism generating poly-Ub chains, which are substrates for PINK1, thereby amplifying mitophagy signals [20, 21] (Fig. 1). However, while the PINK1/Parkin pathway’s role in mitophagy has been studied in the Drosophila model, the mechanisms in the human brain are unclear due to the complexity of mammalian brain structure and function [22–24]. Furthermore, it has been recently confirmed that mitophagy can occur independently of PINK1, Parkin protein, or both, in a variety of tissues [25–27].

#### NIX/Bnip3

B-cell lymphoma 2 kilodalton interacting protein 3 (Bnip3) and its analog NIX are outer mitochondrial membrane (OMM) proteins, which are regarded as apoptotic proteins. Both contain the LC3-interacting region motif (LIR) to facilitate direct interaction of the mitochondria with LC3 or other LC3/GABARAP family members to recruit the autophagosomal machinery (Fig. 1). NIX was initially shown to act as a mitophagy receptor during reticulocyte maturation when mitochondria are eliminated from erythrocytes [28]. NIX/Bnip3 has been confirmed to interact with the homologue of autophagy-related gene protein 8 (Atg8) through its LIR motif, an autophagy protein in the newly formed autophagy membrane that initiates mitophagy [29]. In addition, overexpression of NIX improves the production of ATP in the cells of patients with Parkinson’s disease (PD) [30]. Furthermore, NIX/Bnip3 mediates mitophagy by hypoxia and stabilization of HIF1α [31]. These studies suggest that exploitation of mitochondrial-mediated autophagy mechanisms might be useful for treating disease. NIX/Bnip3-mediated mitophagy has been confirmed to protect against cerebral ischemic injury [17, 32].

#### FUNDC1

FUN14 domain containing 1 (FUNDC1) is an OMM protein that contains a transmembrane LIR motif, which can be activated under hypoxic conditions. During hypoxia-induced stimulation, FUNDC1 dephosphorylation by phosphoglycerate mutase family member 5 phosphatase (PGAM5) promotes its interaction with LC3, inducing mitophagy [33]. ULK1 is upregulated and translocated to fragmented mitochondria upon the induction of mitophagy by either hypoxia or mitochondrial uncouplers. In mitochondria, ULK1 interacts with FUNDC1, phosphorylating it at serine 17, which enhances FUNDC1 binding to LC3 [34].

#### p62/SQSTM1

p62/SQSTM1, a cytoprotein, containing a cytosolute LIR motif protein, was the first mammalian selective autophagy receptor to be described [35, 36]. The LIR motif of P62 interacts with multiple sites on LC3. This interaction raises the possibility that upstream factors, such as ULK1, assemble p62 around the autophagosome formation site prior to the formation of the isolation membrane [37]. In Parkin-mediated mitophagy, Parkin recruits ubiquitin and p62 during mitophagy [38]. Furthermore, the phosphorylation of p62 at the S403 site is required for the promotion of autophagosomal engulfment of ubiquitinated mitochondria, and this p62 phosphorylation depends on the activation of TANK-binding kinase 1 (TBK1), which prevents the phosphorylation of p62 and reduces autophagosomal engulfment during mitophagy [39].

#### Other proteins

In addition to the above receptor proteins, other cytoplasmic receptor proteins, such as NBR1, NDP52, and Optineurin (OPTN), have also been confirmed to mediate the mitophagic process [39–41]. Ubiquilin-2 protein, which can bind polyubiquitinated substrates together through its UBD domain, is transported to a protease through its UBL domain, mediating autophagy [42]. Valosin-containing protein (VCP), a ubiquitin-selective partner protein, participates in the autophagic regulation of stress granule (SG) morphology and composition by interacting with lysosomes in neurodegenerative diseases [43]. Furthermore, FK506 binding protein FKBP8 (also known as KFBP38), an OMM protein, can also recruit LC3A by the LIR motif to induce Parkin-independent mitophagy [44].

### Lipid pathways

#### Cardiolipids

Cardiolipids in the brain consist of a small amount of unsaturated fatty acids that are present in the mitochondrial intimum, requiring three translocases to be exposed to the OMM, and can directly bind to LC3 to mediate mitophagy [45, 46]. In line with this, anti-RNAi injection *in vivo* resulted in knockdown of PLS3, the enzyme responsible for translocating cardiolipin (CL) from the IMM to the OMM with the accompanying phosphorylation of PLS3 at threonine 21, and downregulated mitophagy in the controlled cortical impact (CCI) model [47, 48].

#### Cholesterol and fatty acids

In the presence of PINK1, exogenous steroids and fat can promote PINK1 stability and translocation of the Parkin protein, thus participating in the regulation of mitophagy [49]. Thus, cholesterol and fatty acids may also mediate mitophagy. However, cholesterol exerts a dual effect on PINK1-parkin-mediated mitophagy, by impairing lysosomal clearance of mitophagosomes and promoting a progressive oxidative-induced accumulation of OPTN aggregates that prevent its mitochondrial recruitment despite PINK1/parkin activation [50].

## Mitophagy in chronic diseases of the nervous system

**Fig. 2.**
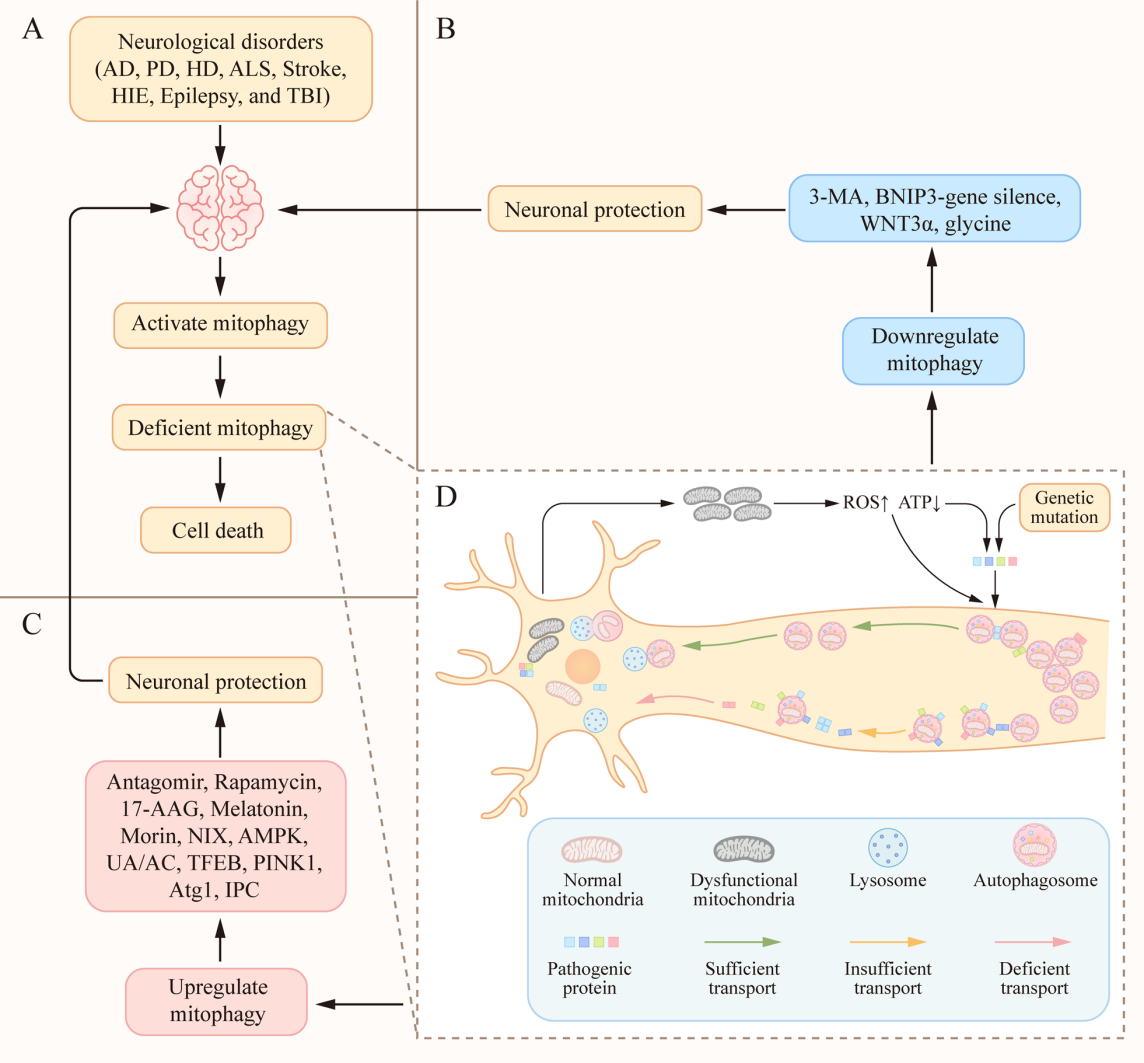
Mitophagy in neurological diseases. **A** The activation of mitophagy is observed in brain tissue after neurological disorders. At the same time, brain injury causes mitochondrial damage and mitochondrial dysfunction, leading to insufficient mitophagy. When mitochondria are damaged, their recyclable energy is exhausted, eventually leading to cell death. **B** Inhibiting mitophagy through the use of 3-MA, BNIP3-gene silencing, WNT3α, and glycine is beneficial for the protection of cranial nerves. **C** In contrast, Promoting mitophagy by the antagomir, rapamycin, 17-AAG, melatonin, morin, NIX, UA/AC, TFEB, PINK1, Atg1, IPC, and AMPK can protect the nervous system. **D** Pathogenic proteins interfere with cargo recognition, cargo loading, and autophagosome trafficking and fusion. Inadequate mitochondrial degradation is responsible for the accumulation of defective mitochondria under these conditions. Mutations in pathogenic proteins reduce mitophagy, resulting in the aggregation of defective mitochondria. The aggregated pathogenic proteins decrease autophagosomes motility in neurons. However, accumulation of pathogenic proteins (i.e., huntingtin, α-synuclein, and p-tau) is often a consequence of defective mitophagy. 3-MA, 3-methyladenine; BNIP3, B-cell lymphoma 2 kilodalton interacting protein 3; UA/AC, urolithinA/actinonin; TFEB, transcription factor EB; PINK1, PTEN-induced putative kinase 1; Atg1, autophagy-related protein; AMPK, AMP-activated protein kinase.

Current research on mitophagy in chronic nervous diseases is mainly focused on neurodegenerative diseases. Studies have shown that some pathogenic proteins are related to neurodegeneration. Genetic mutations can cause accelerated age-dependent generation and self-aggregation of pathogenic protein [51]. On the other hand, mitochondrial dysfunction is a common pathological feature in neurodegenerative disorders which can promotes production and aggregation of pathogenic proteins through excessive oxidative stress and reduced cellular ATP levels [52, 53]. Mutated proteins reduce the mitophagy [41, 54, 55]. Toxicity (such as deficient cargo recognition, inefficient cargo loading, and decreased autophagosome retrograde motility) of pathogenic proteins in the autophagy system can be the primary cause of neurodegenerative disease. However, the accumulation of pathogenic proteins (i.e., huntingtin, α-synuclein, and tau) is often a consequence of defective autophagy [6] (Fig. 2). The pathological characteristic is the mismatch between the accumulation of proteins in affected neurons and damaged cytocells, and protein removal mechanisms, such as mitophagy, resulting in the aggregation of damaged cytoblasts and polyubiquitinated proteins in the cytoplasm and nucleus of neurons [56] (Fig. 2). Mitophagy is stimulated by the accumulation of dysfunctional mitochondria. Changes in the autophagic processes in degenerating neurons can be primary or reactive to other underlying causes. For example, the γ-secretase enzyme Presenilin1 (PS1) carries out cleavage of amyloid precursor protein yielding Abeta peptides, which in various forms have been implicated in AD pathogenesis. Genetic mutation of PS1 inhibits autophagy by reducing vATPase immunoreactivity on lysosomes, elevating lysosomal PH, depressing lysosomal protease activation, and significantly delaying autophagy protein turnover in Alzheimer’s disease (AD) [57]. Using live cell imaging, Maday et al. observed that 80% of autophagosome biogenesis in neurons occurred at the end of the axon and autophagosomes were transported along the axons to dendrites and somatic cells. Autophagosome membranes were also accumulated at the endoplasmic reticulum [8]. The accumulation of pathogenic proteins decreases autophagosome motility in neurons [58–60]. On the other hand, binding of pathogenic proteins to the autophagosome surface or mitochondrial membrane can lead to the accumulation of autophagosomes by reducing their retrograde transport [58–60]. When the nascent autophagosome consumes the target cargo, it merges with the cytosol lysosome and decomposes the target cargo, such as the mitochondria. The membrane anchor protein soluble N-ethylmaleimide-sensitive factor attactment protein receptors (SNAREs) affect the fusion of autophagosomes and lysosomes directly or indirectly by mediating the fusion of organelles or influencing lysosomal biogenesis [61, 62] (Fig. 2). Pathogenic proteins and defective mitophagy increase the accumulation of dysfunctional mitochondria [23, 63–66], thus including a ‘vicious cycle’ reaction. However, the chronological sequence of dysfunctional mitochondria, defective mitophagy and pathongenic proteins is still unclear. Therefore, repairing or enhancing mitophagy might represent a novel treatment for neurodegenerative diseases (Table 1). For example, silomus, also known as rapamycin, has been shown to prevent protein aggregation by inducing mitophagy through direct binding and inhibition of the mammalian target of rapamycin (mTOR) complex (mTORC1) and will also inhibit mTOR complex (mTORC2) activity after prolonged treatment in models of neurodegenerative disease[67–69]; however, its serious side effects, independent of autophagy, are also noteworthy. Furthermore, the focus of treatments for neurodegenerative diseases has shifted away from autophagy towards selective target induction [70].

### Alzheimer's disease

Alzheimer's disease (AD) is the most common neurodegenerative disease and is characterized by a progression from episodic memory problems to severe cognitive decline [71, 72]. The main pathological basis of memory loss is energy deficiency and synaptic inhibition [73, 74]. Biologically, AD is defined by two principal neuropathological hallmarks: the abnormal accumulation of extracellular amyloid-β (Aβ)-containing plaques and intracellular tau-containing neurofibrillary tangles. Converging evidence indicates that Aβ initially accumulates in the medial frontal cortex and medial parietal cortex, which are both elements of the default-mode network (DMN). In contrast, tau is initially deposited in the medial temporal lobe memory system, spreading from the entorhinal cortex to the hippocampus and parahippocampal cortex, and then to other brain regions [75, 76]. Insufficient mitochondrial function and bioenergy output of mitochondrial in AD patients may lead to a reduction in cellular energy levels, concomitant leakage of electrons promotes the formation of reactive oxygen species (ROS), which can damage proteins, membrane lipids and nucleic acids. By initiating membrane lipid peroxidation, ROS generated by mitochondrial dysfunction may also promote the accumulation of pathological extracellular amyloid-β (Aβ) peptides and intraneuronal hyperphosphorylated tau (p-tau) protein and subsequent formation of AD-defining Aβ plaques and neurofibrillary tangles, which in turn promotes mitochondrial defects [77–79]. Direct exposure of synaptic terminals to aggregating Aβ and α-synuclein results in membrane-associated mitochondrial dysfunction, oxidative stress, and impaired glutamate and glucose transport, thereby rendering the synapses vulnerable to excitotoxic degeneration [80, 81], forming a vicious cycle (Fig. 2). Lifestyle interventions (i.e., fasting caloric restriction, and exercise) and Pharmacological agents aimed at improving mitohondrial health and enhancing mitophagy have been evaluated in the AD animal models. Evidence from studies of rodents has shown that fasting and exercise affect signaling pathways in neurons in ways that reduce mitochondrial oxidative stress, stimulate mitochondrial biogenesis, and enhance autophagy [82, 83]. On the other hand, in comparison to non-transgenic (non-Tg) mice, 5xFAD mice, models for mimicking amyloidopathy in AD, manifest prominent synaptosomal mitochondrial dysfunction, and Parkin and LC3BII recruitment in an age-dependent manner, suggesting that synaptosomal mitochondrial deficits are the primary pathology in the Aβ-rich environment, further confirming the relevance of synaptosomal mitochondrial deficits in the development of AD [13]. In the amyloid precursor protein (APP) AD mouse model, both urolithinA (UA) and actinonin (AC), two potent neuronal mitophagy-inducing agents, greatly improved learning and memory retention during the classical Morris water maze (MWM) test. Similarly, in AD induced pluripotent stem cell (iPSC)-derived neurons, mitophagy diminishes AD-related tau hyperphosphorylation and prevents cognitive impairment, suggesting that impaired removal of defective mitochondria is a pivotal event in AD pathogenesis and that mitophagy represents a potential therapeutic intervention target [84].

### Parkinson’s disease

Parkinson’s disease is the second most common neurodegenerative disease after AD, and is characterized by static tremor, bradykinesia, stiffness, and postural instability. By definition, all patients with PD have the neuropathology of PD with the early loss of dopaminergic neurons in the substantia nigra and abnormal deposition of α-synuclein in Lewy bodies, initially in cholinergic and monoaminergic brainstem neurons and in the olfactory system, causing significant synaptic pathology [85–88]. Among the monogenic forms of PD, two genes causing autosomal recessive forms of the disease are PARK2 and PARK6, which encode Parkin and PINK1, respectively, and both play major roles in PINK1/Parkin-mediated mitophagy [89, 90] (Fig. 2). Dysfunctional mitophagy combines with reduced PINK1/Parkin to mediate the pathological mechanisms of PD. However, basal mitophagy in vivo can occur independently of PINK1 in a variety of tissues, as measured using a range of quantitative parameters [25]. Moreover, in the nigral dopaminergic neurons of MPTP mouse model of PD, transcription factor EB (TFEB) overexpression boosts dopamine release in the striatum and probably also triggers the expression of other proteins relevant to the dopaminergic phenotype, which simultaneously drives a neurotrophic effect and gives rise to neuronal cell growth [91].

### Huntington's disease

Huntington's disease (HD) is a neurodegenerative disease caused by an expansion of the cytosine–adenine–guanine (CAG) trinucleotide repeat encoding a polyglutamine (polyQ) tract in the amino-terminal region of Htt protein. Clinically, HD is characterized by psychiatric disorder [92], cognitive decline, and motor dysfunction [93]. At the molecular level, mutant Htt has been reported to affect regulation of the mitophagy system in cellular mechanism [94] (Fig. 2). Extensive accumulation of ubiquitin and p62 is observed in the Htt conditional knockout mouse model, suggesting that Htt protein plays a role in selective autophagy [95]. Mutations in the Htt protein leads to deficient autophagy. It is noteworthy that Htt protein is only partially required for macroautophagy, such as mitophagy, and is not essential for basal autophagy or autophagy induced by nutritional deprivation [70]. Furthermore, the PINK1/Parkin pathway is not affected in the HD animal model, but the fusion of mitochondria with autophagosomes is limited, resulting in damage to the mitophagic mechanism. This damage is partially recovered when PINK1 is overexpressed, thereby improving mitochondrial integrity and protecting neural function [96].

### Amyotrophic lateral sclerosis

Amyotrophic lateral sclerosis (ALS) is a neurodegenerative disease with a pathological basis associated with genetic mutations, and characterized by progressive degeneration of motor neurons in the brain and spinal cord. Mitophagy relies on OPTN, an autophagy receptor, and its kinase TBK1. Importantly, gene mutations reduced OPTN and LC3B recruitment to damaged mitochondria. Damaged OPTN and TBK1 impair the mitophagy mechanism, dysfunctional mitochondria, and protein accumulation. At the same time, other receptors (such as nuclear dot 52 kDa protein [NDP52]) may complement this pathway, because mitophagy is a redundant process involving multiple receptor pathways in the early stages of damage to OPTN. Nevertheless, the decreased efficiency caused by TBK1 or OPTN mutations may be sufficient to lead to enhanced neurodegeneration over time [41, 65, 97] (Fig. 2). Atg1 overexpression significantly extended the life of the ALS transgenic fruit fly model and prevented disease by driving the direct action of the mTOR target Atg1 to positively regulate autophagy [98]. However, therapeutic approaches aimed at enhancing autophagy may not be uniformly beneficial for patients with ALS. More nuanced strategies may be effective, such as enhancing lysosomal function and counteracting the accumulation of autophagosomes or autolysosomes without sufficient degradative ability [99].

## Mitophagy in acute neurological diseases

Compared with chronic diseases of the nervous system, there have been relatively few studies on mitochondrial autophagy in acute diseases of the nervous system. These include studies on non-infectious acute injuries of the nervous system, such as stroke, ischemic and hypoxic brain injury, epilepsy, and traumatic brain injury. The pathological mechanisms are complex and different, but have aspects in common, such as acute injury that activates mitophagy, and an imbalance between autophagy and autophagy flux causes abnormal autophagy mechanisms (Fig. 2). There is still no consensus on whether mitophagy is beneficial or harmful (Table 1). Current literature reports that the reasons for this may be related to factors, such as the differences between in vivo and in vitro studies, the disparity in the periods of disease, crosstalk between mitophagy and apoptosis, and different experimental methods and environments. On the other hand, biological gender differences are important in the brain from a physiological point of view [100]. The dual endothelin-1/vascular signal peptide-activated receptor (DEspR) plays a role in neuroepithelium and neural tube differentiation. DEspR^+/-^ females exhibited better cognitive performance than wild-type females and showed absence of neuropathological changes. This elegant study highlights gender associated cerebral neuronal vulnerability to autophagic dysregulation [101]. However, current research on the gender differences in mitophagy associated with neurological disorders is very limited, which may be a future direction for us. In addition, the detection of autophagy flux—an ongoing autophagic response functionally coupled to lysosomal degradation—may also be an influencing factor in these varied conclusions [102]. The following section elaborates on some acute diseases of the nervous system.

### Stroke

Stroke, also known as "cerebrovascular accident" (CVA), is caused by ischemic or hemorrhagic cerebrovascular disease that can cause severe oxygen–glucose deprivation (OGD) and unrecoverable brain injury. In addition, in ischemic cerebrovascular disease, major brain ischemia for more than a few minutes causes irreversible brain damage to brain cells. Melatonin reduces mitochondrial injury and ROS generation, and inhibits activation of NLRP3 inflammatory entities by upregulating mitophagy related protein (PINK1/Parkin) in rat models of subarachnoid hemorrhage (SAH), thus reducing neuronal cell death, brain edema, and neurodysfunction after SAH [103]. In contrast, in comparison to BNIP3-WT mice, BNIP3-KO mice manifest increased autophagy, decreased apoptosis, and decreased cerebral infarction volume through inhibition of mitophagy by decreasing BNIP3 interaction with LC3, suggesting that BNIP3 gene silencingis beneficial for neuroprotection after stroke [104] (Fig. 2).The differences in these results may be related to factors, such as the constructed stroke animal model, observed post-stroke timing, and undetected autophagy flux.

### Neonatal hypoxic–ischemic encephalopathy

Neonatal hypoxic–ischemic encephalopathy (HIE) refers to hypoxic–ischemic cerebral neuropathy caused by perinatal asphyxia. Severe HIE can lead to cerebral palsy, epilepsy, mental retardation, and cognitive impairment. Brain tissue ischemia causes a large amount of mitochondrial damage. Mitophagy can clear these damaged mitochondria and alleviate brain injury. Bnip3L is a necessary protein involved in initiating mitophagy in cerebral tissue ischemia reperfusion [32]. In models of hypoxic ischemic brain injury in newborn rats, hypoxic ischemia induced excessive autophagy fluxes leading to aggravated brain injury, including increased LC3-II expression, reduction of P62/SQSTM1 protein expression, and decreased cAMP-response element-binding protein (p-CREB). 3-methyladenine (3-MA), an autophagy inhibitor, significantly attenuated the increase in LC3-II and the loss of P62/SQSTM1 and p-CREB, ameliorated neuronal death, improved the results of the MWM test, and ultimately the spatial learning of memory function in rats [105]. In addition, glycine attenuated hypoxia–ischemic injury in neurons or the nervous system by decreasing mitophagy through regulation of the AMPK pathway (such as increasing p62, and decreasing LC3II/I and Bnip3), both in vivo and in vitro [106]. In contrast, ischemic preconditioning treatment significantly increased phosphorylation of AMPK and induced autophagy in the brain, and decreased brain infarct volume, neurological deficits, and neural apoptosis [107]. MiR-30d-5p is one of the members in the miR-30d microRNA (miRNA) family. The miR-30d family regulates a wide range of physiological processes in normal tissues and cancers [108]. In HIE animal models, an increase in miR-30d-5p by agomir (AG) resulted in a reduction of autophagy and an increase in apoptosis, leading to increased infarct volume, delayed recovery of neurological function, and impaired improvement of spatial memory ability. Inhibition of miR-30d-5p by antagomir (AT) enhances autophagy and inhibits apoptosis, contributes to decreased infarct volume, promotes neurological recovery, and improves behavioral performance of rats subjected to HIE [109] (Fig. 2).

### Epilepsy

Epilepsy, although a long-term neurological disease, is often characterized by recurrent acute seizures. The condition has high morbidity and mortality, and seriously impacts the physical and mental health of patients. Autophagy was detected by the autophagy marker protein LC3B and mitochondrial marker TOMM20 in hippocampal tissue samples from patients with refractory temporal lobe epilepsy (rTLE) [110]. In humans, mutations in the gene encoding the mTORC1 autophagy inhibitor are associated with increased susceptibility to epilepsy [111]. In addition, neurotoxicity is also involved in seizures, as excitable toxic substances, such as glutamate act on postsynaptic endings, increasing their depolarization and leading to continuous inflow from synaptic gaps, mitochondrial damage, and the blocking of mitophagy reactions [112–114]. In focal cortical dysplasia (FCD), the common cause of severe childhood epilepsy, over activation of mTOR and p62 accumulation play important roles in the pathogenesis of FCD [115]. Rapamycin, an inducer of mitophagy and inhibitor of mTOR, can prevent the further development of epilepsy in the early periods, whereas late treatment reduces seizure frequency in mice that already have epilepsy [116, 117] (Fig. 2).

### Traumatic brain injury

Traumatic brain injury (TBI) is a traumatic structural injury and/or brain dysfunction caused by external forces. In the cerebrospinal fluid of children with TBI, the autophagy markers Beclin and p62 are increased, and autophagy flux is decreased, suggesting that the increase in autophagy may be associated with a decrease in autophagy flux [118]. It is worth noting that cerebrospinal fluid does not directly reflect autophagy in brain cells. The use of morin, a natural polyphenol, to enhance autophagy, can reduce levels of inflammation markers (TNF-α, IL-6) and apoptosis, and improve memory injury in mild traumatic brain injury [119] (Fig. 2). In line with this, upregulation of nix can decrease neuronal apoptosis and brain water content by increasing mitophagy of TBI rat model [120]. In the rat model of controlled cortical impact (CCI), a TBI model, enhancing autophagy using the experimental cancer drug 17-allylamino-demethoxygeldanamycin (17-AAG) reduced the brain water content and neuronal death, and promoted the recovery of motor function [121]. In contrast, by inhibiting autophagy and apoptotic cell death using nasal WNT3α therapy in a TBI mouse model, mice showed reduced overall death of neurons compared with controls [122].

## Conclusions

Most of the work on mitophagy in the nervous system has focused on its pathogenesis at the molecular level and potential targets for the treatment of neurological diseases. However, there is still controversy surrounding the exact mechanisms of the receptor-mediated autophagy pathway, differences in research and environmental conditions, whether targeting mitophagy for disease treatment is more harmful than beneficial, and how best to address these issues. In addition, there are inconclusive results regarding the targeting of mitophagy in long-term chronic diseases of the nervous system, and a lack of extensive and rigorous research in short-term acute diseases of the nervous system. These issues need to be addressed by future studies.

## Data Availability

Not applicable.

## Abbreviations

CMA: Chaperone-mediated autophagy
LAMP2: Lysosome-associated membrane protein 2
ATP: Adenosine Triphosphate
Bnip3L: BCL/adenovirus E1B interacting protein 3-like
PTEN: Phosphase and tensin
PINK1: PTEN-induced putative kinase 1
LC-3: Light chain 3
IMM: Inner mitochondrial membrane
p-Ub: Phosphorylation of ubiquitin
Bnip3: B-cell lymphoma 2 kilodalton interacting protein 3
OMM: Outer mitochondrial membrane
LIR: LC3-interacting region motif
SNAREs: Soluble N-ethylmaleimide-sensitive factor attactment protein receptors
p-tau: Hyperphosphorylated tau
ROS: Reactive oxygen species
iPSC: Induced pluripotent stem cell
PS1: Presenilin1
PD: Parkinson’s disease
FUNDC1: FUN14 domain containing 1
PGAM5: Phosphoglycerate mutase family member 5 phosphatase
TBK1: TANK-binding kinase 1
OPTN: Optineurin
VCP: Valosin-containing protein
SG: Stress granule
AD: Alzheimer’s disease
non-Tg: Non-transgenic
APP: Amyloid precursor protein
UA: UrolithinA
AC: Actinonin
MWM: Morris water maze
SNpc: Substantia nigra pars compacta
DA: Dopamine
LBs: Lewy bodies
TFEB: Transcription factor EB
HD: Huntington's disease
CAG: Cytosine–adenine–guanine
polyQ: Polyglutamine
ALS: Amyotrophic lateral sclerosis
NDP52: Nuclear dot 52 kDa protein
DEspR: Dual endothelin-1/vascular signal peptide-activated receptor
CVA: Cerebrovascular accident
OGD: Oxygen–glucose deprivation
SAH: Subarachnoid hemorrhage
HIE: Hypoxic–ischemic encephalopathy
3-MA: 3-methyladenine
AG: Agomir
AT: Antagomir
rTLE: Refractory temporal lobe epilepsy
FCD: Focal cortical dysplasia
TBI: Traumatic brain injury
CCI: Controlled cortical impact
AAG: 17-allylamino-demethoxygeldanamycin
